# Presenilin 1 transgene addition to amyloid precursor protein overexpressing transgenic rats increases amyloid beta 42 levels and results in loss of memory retention

**DOI:** 10.1186/s12868-016-0281-8

**Published:** 2016-07-07

**Authors:** Cansu Agca, Diana Klakotskaia, Todd R. Schachtman, Anthony W. Chan, James J. Lah, Yuksel Agca

**Affiliations:** Department of Veterinary Pathobiology, College of Veterinary Medicine, University of Missouri, 1600 East Rollins Street, Room W191, Columbia, MO 65211 USA; Department of Psychological Sciences, University of Missouri, Columbia, MO 65211 USA; Yerkes National Primate Research Center, Emory University, Atlanta, GA 30329 USA; Department of Neurology, Center for Neurodegenerative Disease, Emory University, Atlanta, GA 30322 USA

## Abstract

**Background:**

We previously reported the production of transgenic rats (APP21 line) that over-express human amyloid precursor protein (APP) containing Swedish and Indiana mutations. In order to generate a better model for Alzheimer’s disease (AD), the APP21 rat line was used to generate double transgenic line that over-expressed Presenilin 1 (PS1) with L166P mutation in addition to APP transgene (APP + PS1 line).

**Results:**

Thirty-two double transgenic founders were generated and the ultimate transgenic founder was selected based on PS1 transgene copy number and level of amyloid-beta (Aβ)_42_ peptide. The APP + PS1 double transgenic rats had 38 times more PS1 in brains compared to APP rats. Behavioral assessment using Barnes maze showed that APP + PS1 rats exhibited a larger learning and memory deficit than APP21 rats. Double transgenic rats also produced more Aβ_42_. Histological examination of the brains showed that the APP21 rat line displayed neurofibrillary tangles and in contrast, the APP + PS1 line showed chromatolysis in hippocampal neurons and neuronal loss in CA3 region of hippocampus.

**Conclusions:**

Due to the separate segregation of APP and PS1 transgenes in APP + PS1 double transgenic rats, this transgenic line may be a valuable model for studying the effects of various levels of APP and PS1 transgenes on various aspects of brain pathologies associated with the AD phenotype.

**Electronic supplementary material:**

The online version of this article (doi:10.1186/s12868-016-0281-8) contains supplementary material, which is available to authorized users.

## Background

Alzheimer’s disease (AD) is one of the most devastating and costly diseases that affect approximately 12 % of the population over the age of 65 [2]. Alzheimer’s disease is associated with the over-production and reduced clearance of amyloid-beta (Aβ) peptides [19]. Cleavage of amyloid-β precursor protein (APP) by the presenilin (PS) 1 enzyme results in the release of Aβ peptides, which aggregate and form Aβ plaques in the brain. These Aβ plaques and neurofibrillary tangles are the two primary pathological manifestations of AD in the brain. Amyloid peptides are produced via the cleavage of APP by ϒ-secretase, a protease complex of four proteins that includes PS1 or PS2, which are both homologous proteins that contain the catalytic site of the enzyme [7]. ϒ-secretase cleaves the β-amyloid protein into two major forms of Aβ polypeptides, Aβ_40_ and Aβ_42_. The relative amount of the longer form of Aβ, Aβ_42_, is particularly critical for AD progression because it is more prone to aggregation than the shorter Aβ_40_ peptide [4, 10, 23]. In addition, the ratio of Aβ_42_/Aβ_40_ correlates with the load of Aβ plaques in the brain [8, 14]. Furthermore, the reduction of PS1 mRNA using siRNA against PS1 reduced Aβ_42_ in cultured cells [11] which suggests that lower levels of PS1 lead to decreased production of Aβ_42_.

We previously generated rats that overexpress human APP containing the Swedish and Indiana mutations [1]. The APP21 line expressed high levels of APP, but failed to produce Aβ plaques in the brain. However, intrahippocampal seeding of dilute Alzheimer brain extracts containing aggregated Aβ led to the production of plaques in APP21 rats [20]. This finding indicates that plaque formation depends on environmental challenges, conditions which laboratory rats are not exposed to routinely. In order to increase the pathogeny of overexpressed APP, we generated double transgenic rats from the APP21 line that over-express both the PS1 and the APP transgenes. Our hypothesis was that the addition of a PS1 transgene would increase more toxic Aβ_42_ polypeptide and thus, would produce a more suitable model for examining AD.

## Methods

### Animals, construct design, and preparation of vesicular stomatitis virus glycoprotein pseudotyped lentivirus

The homozygous APP21-transgenic rat line [1], containing human APP with the Swedish double missense mutation (K595M/N596L) and Indiana single missense mutation (V642F), were used for the generation of APP-PS1 double transgenic rats. Inbred Fischer 344 rats were used as the background strain for the APP21 rat line. The human PS1 cDNA coding region corresponding to 285-1688 bases of PS1 variant 1 (GenBank Accession number NM_000021.3) with L166P mutation was cloned into the Lentivial vector (pLV) containing ubiquitin-C (Ubi-C) and enhanced green fluorescence protein (eGFP; pLVU-eGFP) cassette [17] in place of the eGFP coding sequence. The new vector was designated as pLVU-PS1. The Ubi-C promoter, which is upstream of PS1, controlled PS1 transcription. The pLVU-PS1 was a self-inactivating vector, composed of the woodchuck hepatitis virus post-transcriptional regulatory element (WRE) to increase transcription levels and minimize position effects. Additionally, a human immunodeficiency virus-1-flap element was inserted between the 5′ long terminal repeat and the internal promoter, which increased the titer. The preparation of vesicular stomatitis virus glycoprotein pseudotyped Lentivirus was as described by Lois et al. [17]. All animal studies were performed in accordance with the University of Missouri’s Animal Care and Use Committee guidelines and the Institute for Laboratory Animal Research Guide for the Care and Use of Laboratory Animals. The rats were housed in conventional cages at 20–25 °C in a controlled lighting environment and provided free access to water and standard pellet rodent chow.

### Zygote collection, microinjection of lentiviral vector, and embryo transfer

Homozygous APP21-transgenic female rats (28–30 day-old) were super ovulated by administering intraperitoneal (IP) injections of 15 IU pregnant mares’ serum gonadotropins (PMSG) followed by IP injections of 15 IU human chorionic gonadotropin 52 h after PMSG. Twenty-four hours after mating, rats were euthanized using CO_2_ and zygotes were collected from the oviducts. Morphologically normal zygotes having two pronuclei and a sperm tail were used for lentiviral vector injections into perivitelline space as described earlier [1]. Lentiviral vector-injected zygotes were then transferred into the oviducts of 8–10 week-old Sprague–Dawley pseudopregnant recipient rats.

### Genomic DNA isolation and polymerase chain reaction

Genomic DNA from tail-snips was isolated using the Wizard Genomic DNA Purification Kit (Promega, Madison, WI). Screening of transgenic rats was conducted by PCR using primers annealing the Ubi-C promoter (Forward; TGTCCGCTAAATTCTGGCCGTT) and PS1 transgene (Reverse; AGCATTCAGAATTGAGTGCAGGGC). The PCR reactions were carried out using 50 ng genomic DNA, 100 ng of each primer and 1.5 U Biolase taq (Bioline, Randolph, MA), and amplified PCR products were size separated through a 1 % agarose gel stained with ethidium bromide for visualization.

### ELISA measurement of Aβ_40_ and Aβ_42_

Serum samples were analyzed for Aβ_40_ and Aβ_42_ using commercial ELISA kits (Genetics Company, Schlieren, Switzerland). Serum samples were diluted in assay buffer and processed according to the manufacturer’s recommended protocols. Briefly, samples and standards were incubated in capture wells overnight at 4 °C with either a biotinylated Aβ_40_ or an Aβ_42_-specific antibody. After several rinses, the enzyme-conjugated detection reagent was added to the wells for 30 min. After additional rinses, wells were incubated with the chromogen solution for 30 min at room temperature and were shielded from light. After the addition of the stop solution, the wells were read for absorption at 450 nm and the Aβ concentration in the samples was calculated based on standard curves.

### Southern blot analysis

Southern blot analysis was conducted to determine the copy number of the transgene in founder rats. Genomic DNA was digested with EcoRI, which cut the junction of the PS1 and the WRE transgene. The digestion products were size-separated through a 0.8 % agarose gel and transferred to a Genescreen plus membrane (Perkin Elmer, Wellesley, MA) overnight. The 524 bp PS1 probe template was prepared by amplification of pLVU-PS1 using forward (AGGTCCACTTCGTATGCTGGTTGA) and reverse (TGATGGAGATTGGAAGAGTGGCA) primers. The ^32^P labeled probe was generated using the probe template, Ready-To-Go DNA Labeling Beads (Amersham Biosciences, Piscataway, NJ) and [α-^32^P]-dCTP (Perkin Elmer, Wellesley, MA). The membranes were prehybridized in 10 % dextran sulfate, 6 × saline sodium sitrate (SSC), 1 % sodium dodecyl sulfate (SDS) for 2 h and hybridized using the ^32^P labeled probe overnight before exposure to BioMax MS-1 Autoradiography Film (Kodak, Rochester, NY).

### Northern blot analysis

Total RNA was isolated using Trisure (Bioline, CA) from founder APP21 + PS1 and APP21 transgenic rats. Brain, heart, kidney, liver, and lung tissues were used in Northern blot analysis. Total RNA was size-separated through 1 % agarose gel before transferring to the Genescreen plus membrane overnight. The membrane was prehybridized in 10 % dextran sulfate, 5 × SSPE, 50 % formamide, 5 × Denhardt’s, 1 % SDS at 42 °C for 6 h prior to hybridization with the PS1 probe overnight. After exposure to BioMax MS-1 Autoradiography Film, membranes were stripped to remove labeled cDNA probe by boiling for 1 h in 1 %SDS and 0.1× SSC. The intensity of the bands was determined using Kodak 1D v 3.6.3 software (New Haven, CT).

### Behavioral measures

Behavioral assessment was conducted using APP + PS1 (n = 12), APP (n = 11), and non-transgenic Fischer 344 (n = 12) rats when their average age was approximately 10 months. Prior to behavior studies, each rat was handled at least 3 times to reduce stress due to handling during the behavioral testing.

#### Barnes maze

The maze consisted of a grey circular platform 122 cm in diameter. The platform was surrounded by a wall that was 30.5 cm in height. The maze was elevated 83.8 cm above the floor by a stand. Twenty holes measuring 10.2 cm in diameter were evenly spaced around the perimeter. A rectangular grey escape box (28 cm in length × 12.7 cm wide × 7.6 cm high at the area closest to the maze tapering to 16.5 cm high) could be placed beneath any hole. The escape box included an entry ramp that provided easy entry access for the rat. Black curtains were hung around the maze and above the maze walls to surround the apparatus and ensure that rats could only use the visual cues provided in the maze, rather than the distal cues within the testing room. Proximal cues were more likely to remain constant, within subjects and across subjects, during the course of training. Four visual cues consisting of various shapes (triangle, square, circle, cross) were placed at evenly spaced intervals on the inside of the maze walls. Two 86-W, 120-V floodlights producing 1690 lumins were hung above the platform served to brightly light the maze in order to create a potentially aversive environment to help motivate the rats to escape from the brightly lit, open surface in favor of the dark environment of the escape box. One light hung 68.5 on side of the maze while the other hung 137 cm from the other side.

Each rat was assigned an escape hole number under which the escape box was placed on each test trial; assigned hole numbers were alternated across rats to eliminate odor cues for consecutively tested rats. The escape box location remained constant for any individual rat across test trials. Behavioral testing consisted of two pretraining trials on the first day, and 6 evaluation trials (2 trials/day) over a period of 3 days. Each day, the animals were transferred in their home cage from their colony room to the testing room 30 min prior to the start of testing. On the pretraining trial, the rat was preexposed to the goal box (with the goal box placed in the same hole that would be used during subsequent training) by placing the rat in the goal box for 90 s. The rats’ escape from the goal box was prevented by covering the opening to the maze surface with a grey, opaque start box (a 23 cm × 23 cm box). A training trial began by placing the rat under the start box positioned in the center of the platform. After 30 s, the box was lifted and the rat had a maximum of 5 min (300 s) to locate and enter the escape box. Latency (time it took for the rat to find and enter the escape box) and total errors (nose-pokes into non-escape holes) were recorded. If the rat did not enter the escape box within 5 min, it was gently guided there by the experimenter’s hand. After 30 s, the rat was removed from the escape box and returned to its home cage. Rats were allowed to rest in their home cage in the testing room for 30 min before starting their second daily trial. The platform and escape box were cleaned after every trial with a 20 % ethanol solution. After the third day, testing abated for 3 days, after which retention was evaluated for two additional trials (1 day), conducted exactly like those just described. The following day, the rats were given reversal training in which the rats were given training exactly like those of original acquisition, except that the escape box was located in a new location (in the opposite quadrant). Reversal training continued for a total of 3 days.

### Histopathology

Nine of the female rats [3 of each: APP + PS1, APP, and wild type (WT)] were euthanized at 18–19 months of age and their brains were collected. Tissues were fixed in 10 % formalin for 48 h prior to paraffin embedding. Hematoxylin and eosin staining was used to determine histological changes. The stained slides were analyzed using a Nikon Eclipse E600 microscope (Melville, NY) and an Olympus DP72 Camera (Center Valley, PA).

### Statistical analysis

Statistical analysis for PS1 mRNA expression, serum Aβ level comparisons, and behavioral analyses were performed using general linear models in SAS (SAS 9.3, SAS Institute Inc., Cary, NC, USA). For the Barnes maze, the average of each animal’s daily score was used in the analysis of overall performance during acquisition and reversal training. Comparisons of daily performance were analyzed separately and differences were considered significant at *p* < 0.05 for all analyses.

## Results

### APP21-PS1 double transgenic founders

**Fig. 1:**
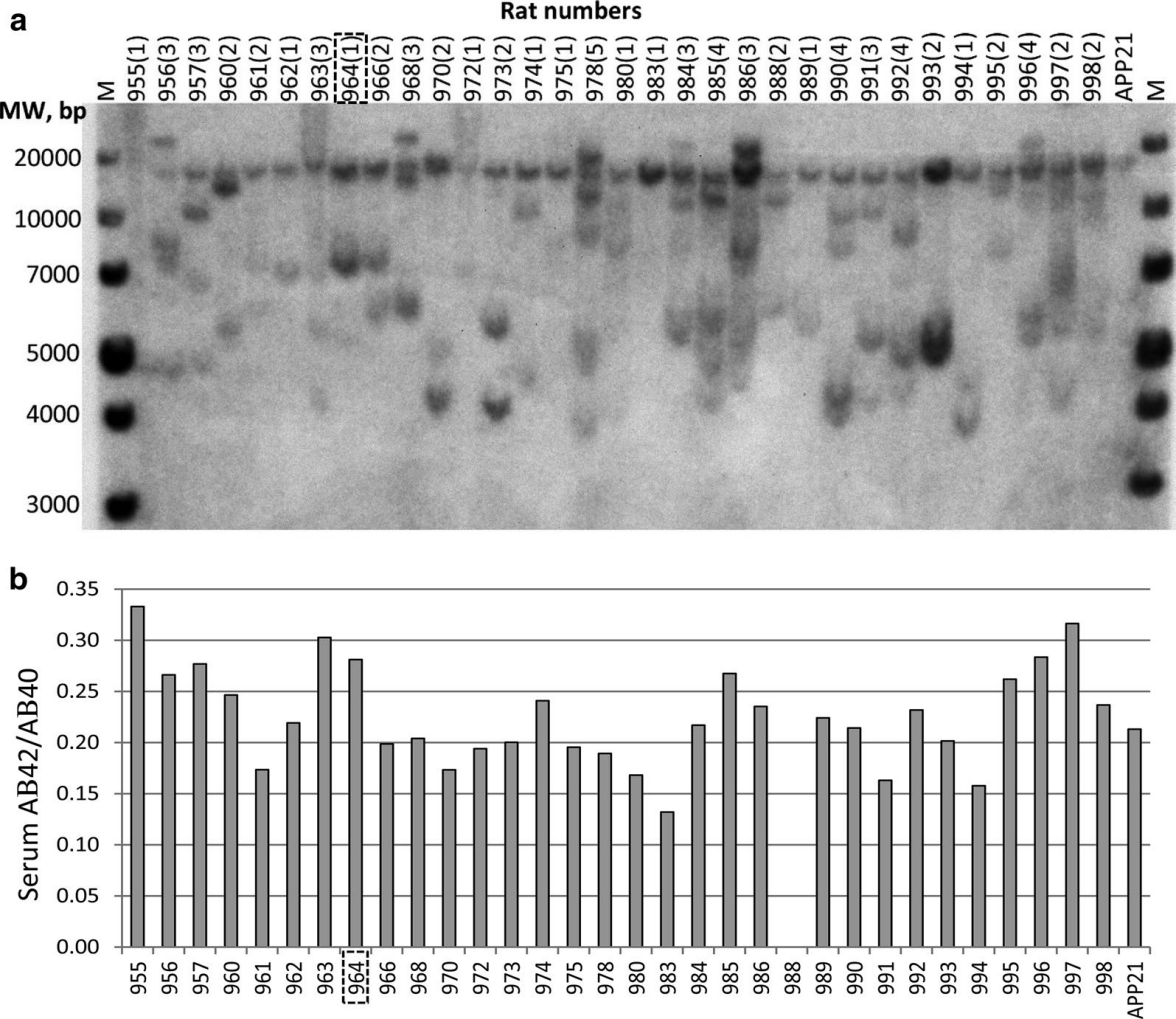
**a** Southern blot analysis of the genomic DNA of the founder animals used to determine the copy number of PS1 transgene. The copy number of the PS1 transgene was determined by the number of hybridized genomic DNA segments in the autoradiogram and is shown in parenthesis above the rat numbers. M represents the molecular weight (MW) markers. **b** Serum Aβ_42_/Aβ_40_ levels of APP + PS1 double transgenic founder animals and APP21 rats at 8 weeks of age. The Aβ_42_/Aβ_40_ ratio for rat 988 could not be determined because its Aβ_42_ level was lower than the detection limit of the test. Founder 964 was selected based on having one PS1 transgene and high Aβ_42_/Aβ_40_ levels as well as for having a large litter with high Aβ_42_/Aβ_40_ levels

Forty-five rats were born from the lentiviral vector which contained PS1 transgene-injected embryos and 32 of them were PCR positive for the PS1 transgene. Southern blot results showed that transgene copy number ranged between 1 and 5 for each PS1 transgenic founder rat (Fig. 1a). Serum Aβ_42_ ranged between 25 and 479 pg/ml in the founder animals at 4 weeks of age and transgene copy number positively correlated with serum AB42 levels in founder animals (r = .54). Serum Aβ_40_ and Aβ_42_ levels at 8 weeks of age were also determined in founder animals (Fig. 1b). Due to the segregation of multiple integration sites in subsequent generations of transgenic animals, founder animals with one copy transgene were used for breeding. Founders with one PS1 transgene copy and relatively high serum Aβ_42_ levels were considered in order to determine the ultimate founder animal. Founder 964 had one of the highest serum Aβ_42_ (196 pg/ml) levels as well as Aβ_42_/Aβ_40_ levels (0.28) at 8 weeks of age. Furthermore, the F1 generation from founder 964 had the highest serum Aβ_42_ levels and had a relatively large-sized litter. Thus, founder 964 was used as the transgenic founder for generation of APP + PS1 transgenic rats.

**Fig. 2:**
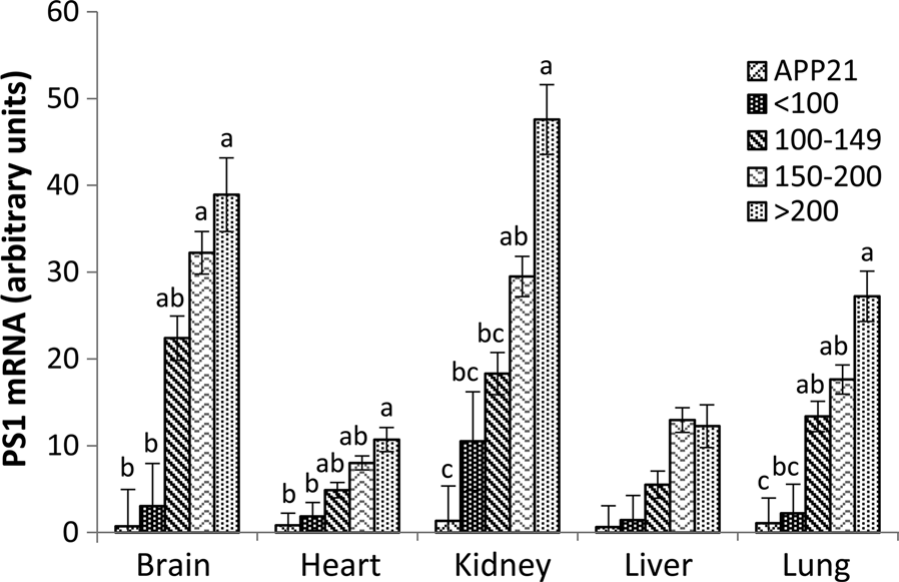
PS1 expression in founder animals and APP controls as determined by Northern blot analysis. The expression of PS1 was analyzed by combining animals with similar serum Aβ_42_ levels and statistical analysis conducted for each tissue. The *figure legend* indicates the range of serum Aβ_42_ level (pg/ml) of the founder animals or the APP control rats. *Different letters* above *each bar* indicates significantly different (*p* < 0.05) expression

Northern blot analysis of various tissues from the founder APP + PS1 transgenic animals showed that brain, heart, kidney, liver, and lung had 38, 8, 19, 15, and 15 fold more PS1 mRNA (P < 0.01), respectively, compared to the APP21 rat line. Serum Aβ_40_ and Aβ_42_ levels at 8 weeks were positively correlated with PS1 mRNA levels in APP + PS1 founders (Table 1). Statistical analysis of the PS1 expression of founder animals was done by grouping the animals based on their 8 wk serum Aβ_42_ levels. The groups comprised of rats with Aβ42 serum levels of less than 100 pg/ml (<100), between 100 and 149 pg/ml (100–149), between 150 and 200 pg/ml (150–200), and greater than 200 pg/ml (>200), as well as APP21 rats that did not have the PS1 transgene. PS1 expression in tissues increased significantly *(p* < 0.05) as serum Aβ_42_ levels increased (Fig. 2; Additional file 1: Table 1) with the exception of the liver, in which PS1 levels were not significantly different.

**Table 1:** Correlation coefficients of serum Aβ_42_ and Aβ_40_ levels to PS1 mRNA levels of the organs

PS1 mRNA	Serum Aβ_42_	Serum Aβ_40_
Brain	0.49	0.36
Kidney	0.48	0.30
Heart	0.60	0.34
Liver	0.45	0.22
Lung	0.54	0.31

### Subsequent generations of APP + PS1 double transgenic rat line

The APP21 line has been previously bred as a homozygous transgenic line. However, homozygous APP + PS1 rats failed to produce pups. Thus, the APP + PS1 double transgenic line was retained as homozygous for APP transgene and hemizygous for PS1 transgene.

**Fig. 3:**
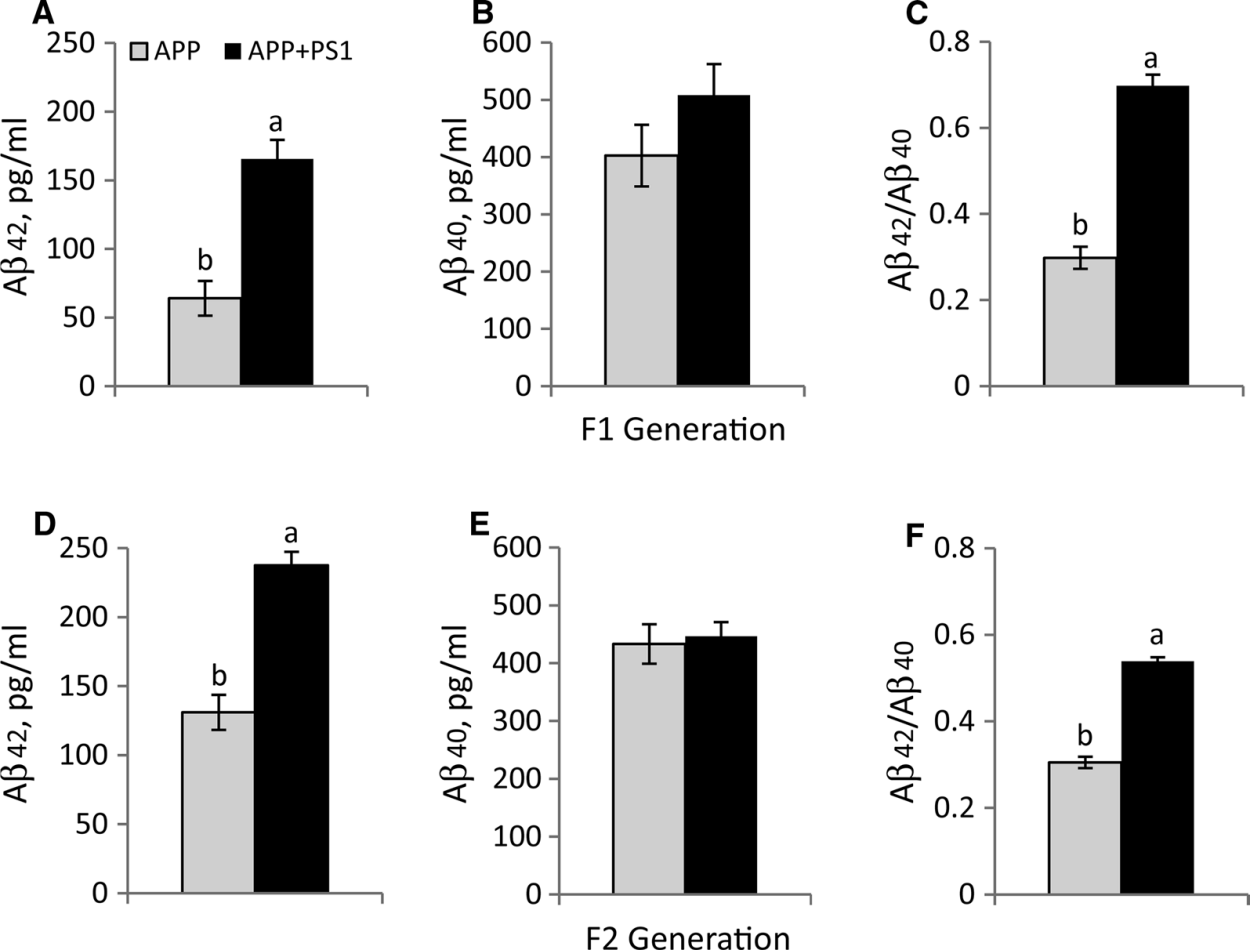
Serum Aβ_42_ (**A, D**), Aβ_40_ (**B, E**) and Aβ_42_/Aβ_40_ (**C, F**) levels of 30 day old F1 and 50 day old F2 generations. *Different letters* above *each* *bar* indicate significantly different levels of Aβ_42_ and Aβ_42_/Aβ_40_

Serum Aβ_40_ and Aβ_42_ levels were determined in F1 generation pups at 30 days of age and in F2 generation pups at 50 days of age (Fig. 3; Additional file 2: Table 2). The Aβ_42_ levels increased with age in both APP and APP + PS1 lines and both F1 and F2 generation APP + PS1 rats had significantly greater (*p* < 0.05) Aβ_42_ levels compared to APP rats. Serum Aβ_42_/Aβ_40_ levels were approximately two times greater in the APP + PS1 rats compared to the APP rats for both F1 and F2 generations (*p* < 0.001), but serum Aβ_40_ levels were similar between PS1 + APP and APP rats and did not change with age.

### Behavior changes among AP + PS1, PS and fischer wild type rats

#### Barnes maze

There were no statistical differences in performance between male and female rats, thus the data were collapsed over sex for all of the behavioral analyses.

##### Acquisition

**Fig. 4:**
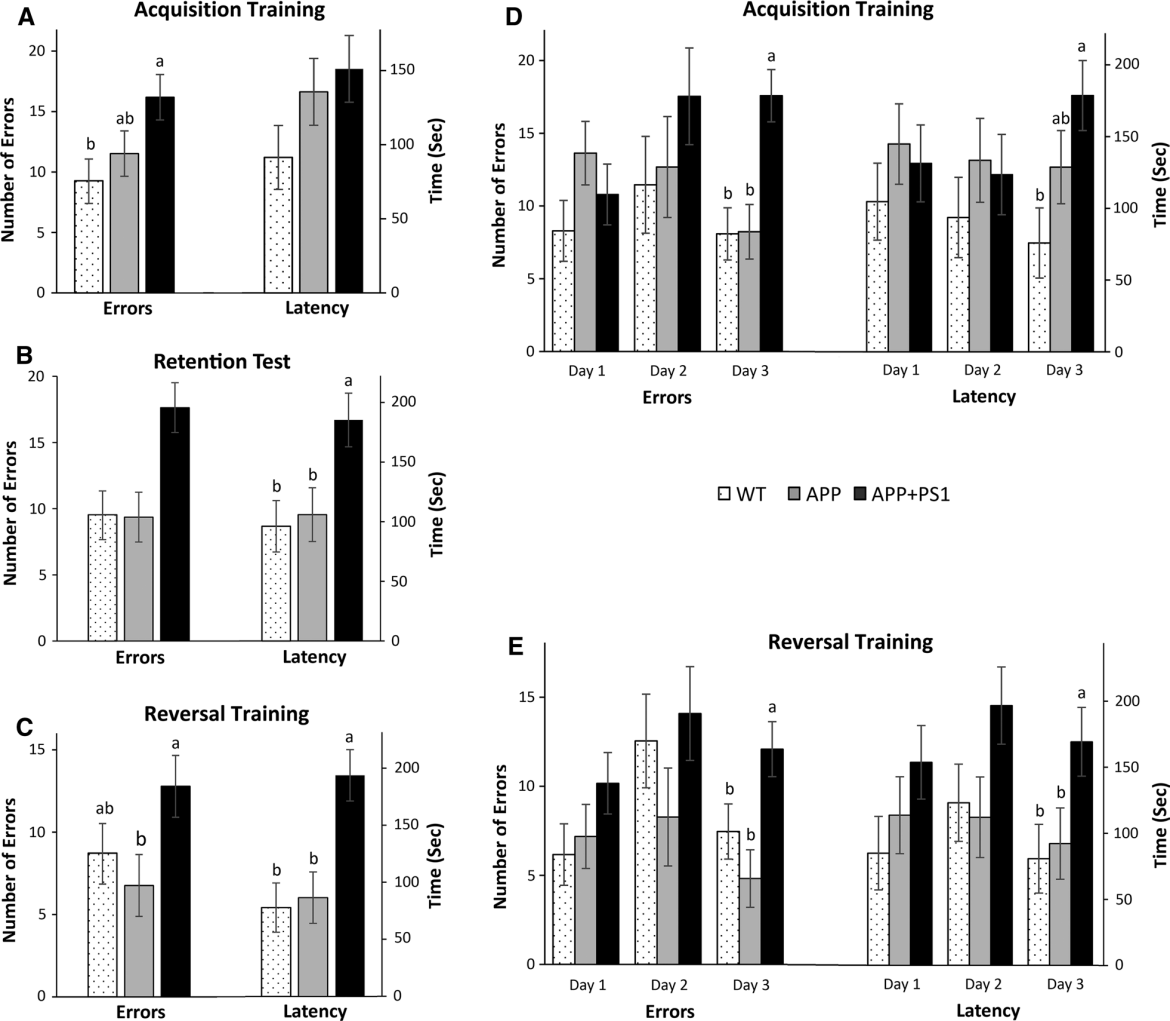
Overall performance of Barnes maze Acquisition Training (**A**) Retention (**B**), and Reversal Training (**C**). Daily performance of Acquisition Training (**D**), and Reversal Training (**E**). Data is expressed as group mean ± SEM. *Different letters* above *each bar* indicates significantly different *(p* < 0.05) expression

Overall, APP + PS1 rats made significantly more errors compared to WT rats (*p* = 0.04) during acquisition training, but there were no group differences in latency (*p* > 0.05; Fig. 4a). More specifically, there were no statistical differences in performance among the groups on acquisition days 1 and 2 (*p*s > 0.05), however, on day 3, APP + PS1 rats made significantly more errors compared to both APP and WT rats (*p* < 0.001) and had significantly longer latencies compared to WT rats only (*p* = 0.02; Fig. 4d). Overall, these results suggest that the APP + PS1 rats had a larger learning deficit in the Barnes maze task than the APP rats.

##### Retention

After the retention interval, APP + PS1 rats made numerically more errors (*p* = 0.1) and had significantly longer latencies (*p* = 0.01) than both the APP and WT rats (Fig. 4b), implying a larger memory deficit in the APP + PS1 rats. These differences in memory retention may be due to the larger learning deficit in APP + PS1 animals during acquisition training.

##### Reversal

Overall, APP + PS1 rats made significantly more errors (*p* = 0.04) and had significantly longer latencies (*p* = 0.03) compared to both the WT and APP rats during reversal training (Fig. 4c). More specifically, there were no significant differences on days 1 and 2 of reversal training (*p*s > 0.05), but on day 3, APP + PS1 rats made significantly more errors (*p* < 0.01) and had significantly longer latencies (*p* = 0.04) than both the APP and WT rats (Fig. 4e). These results suggest deficits in reversal learning in the APP + PS1 rats. Additional file 3: Table 3 contains the dataset of the Barnes Maze.

### Histopathology of brain

**Fig. 5:**
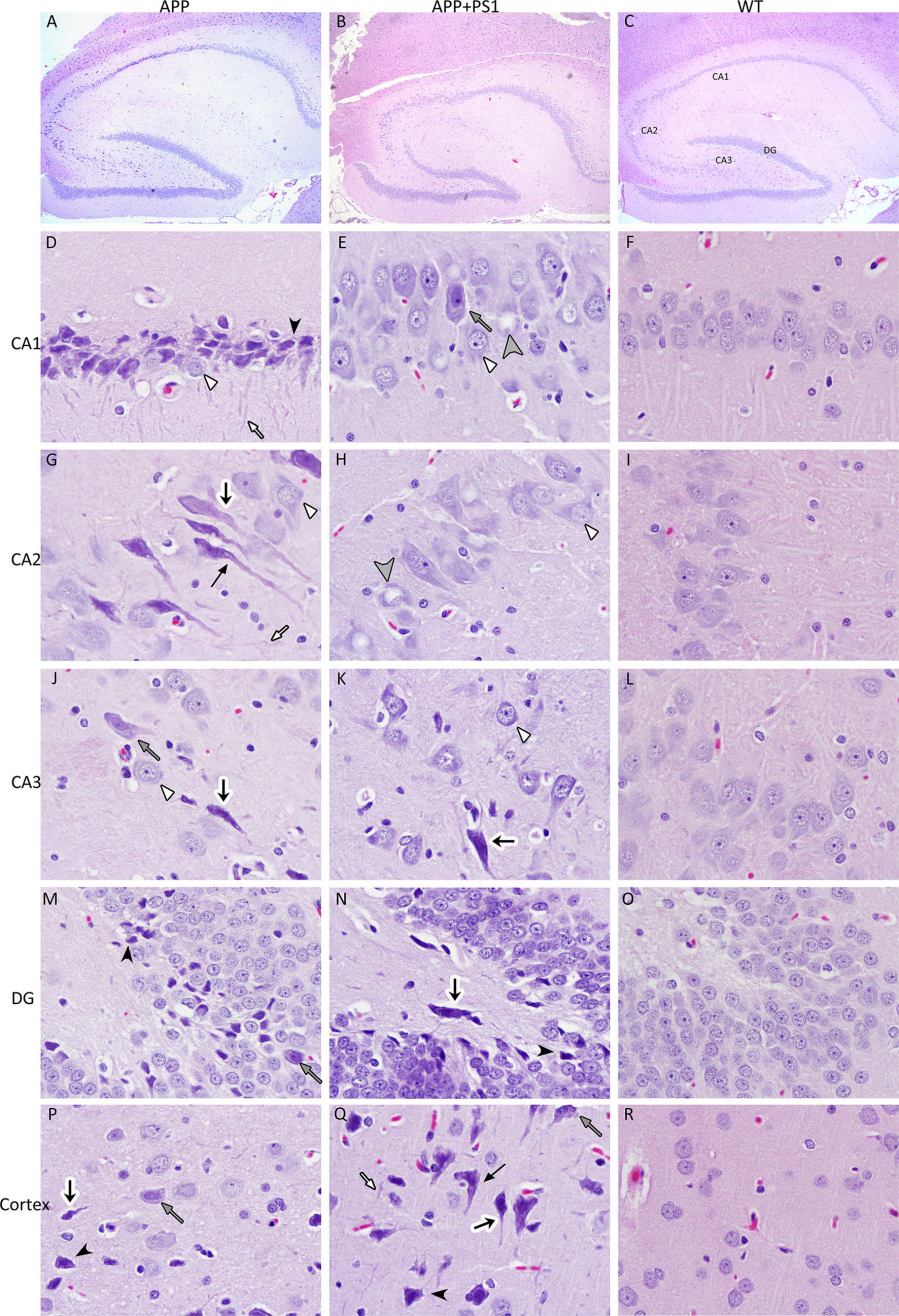
Hematoxylin and Eosin staining of APP, APP + PS1, and WT rat brains. 4× magnification of a hippocampus section from an APP (**a**), APP + PS1 (**b**), and WT rat (**c**). CA1, CA2, CA3 regions of the hippocampus and dentate gyrus (DG) are labeled in **c**. Panels **d**–**r** show 60X magnification of the brain and *each arrow* style represents similar pathology throughout the figure. **d** APP CA1 region with necrotic and *dark colored* pyramidal neurons (*black arrowhead*), ghost tangles (*white arrow*), and normal looking neurons (*white arrow head*). **e** APP + PS1 CA1 showing degeneration with condensed Nissl substance (*grey arrow*) and chromatolysis (*grey arrow head*). **f** WT CA1. **g** APP CA2 with neurofibrillary tangles in viable neurons with visible nuclei (*black arrow*) and non-viable neurons (*black arrow* with *white outline*) and ghost tangles. **h** APP + PS1 CA2 showing chromatolysis in neurons. **i** WT CA2. **j** APP CA3 with neurofibrillary tangles and condensed Nissl substance. **k** APP + PS1 CA3 with neurofibrillary tangles. **l** WT CA3. **m** APP DG granule cells with condensed Nissl substance and necrotic cells. **n** APP + PS1 DG with necrotic cells and neurofibrillary tangles. **o** WT DG. **p**, **q** APP and APP + PS1 cortex neurons with condensed Nissl substance, neurofibrillary tangles, necrotic neurons, and ghost tangles. **r** WT cortex

The hippocampal pyramidal cell layer of *Cornu Ammonis* (CA) 1 and CA2 regions of the APP transgenic rats contained many necrotic neurons and neurofibrillary tangles (Fig. 5a, d, and g). Mineralization was also evident as indicated by the rough cell membrane observed in some of the neurons in the hippocampus and the cortex in both the APP and the APP + PS1 rats. In addition to neurons containing flame-shaped neurofibrillary tangles, hippocampus also contained remnants of neurofibrillary tangles that were embedded in the neuropil (ghost tangles) in the APP rats (Fig. 5d, g). Neurofibrillary tangles were present in viable cells that had nuclei as well as neurons with no visible nuclei (Fig. 5). The CA3 region also contained necrotic neurons and neurofibrillary tangles in both the APP and APP + PS1 rats (Fig. 5j, k).

Both the APP and APP + PS1 rats had fewer pyramidal neurons in the CA3 region compared to WT, but the APP + PS1 animals had more severe neuronal loss in the CA3 region (Fig. 5b). The hippocampal pyramidal cell layer in the APP + PS1 rats had many neurons with central and segmental chromatolysis, showing eccentric displacement of the nucleus and loss of Nissl substance with the exception of the margins of the cell body (Fig. 5h, k). Both the APP and the APP + PS1 brain cortex contained neurons with various degrees of degeneration that ranged from neurons with condensed Nissl substance to dark necrotic neurons and ghost tangles (Fig. 5p, q). The brain cortex of the APP + PS1 animals had larger areas with dark stained neurons, which indicated more severe neuronal necrosis (Fig. 5q).

## Discussion

The production of transgenic rats using lentiviral vectors is an efficient method for gene transfer. However, the major drawback of lentiviral vectors is the integration of the transgene into multiple sites of the genome. Multiple copies of the transgene result in high levels of expression, but segregation in subsequent generations drastically decreases transgene expression. In this study, the founder animal was selected among the animals that had only one copy of the transgene and those that also had the greatest serum Aβ_42_ levels and Aβ_42_/Aβ_40_ ratio.

Serum Aβ_42_ and Aβ_40_ levels were positively correlated with PS1 mRNA expression, indicating that the addition of the PS1 transgene contributed to the production of both Aβ_42_ and Aβ_40_. Furthermore, the greater correlation efficiencies for Aβ_42_ and PS1 mRNA expression indicate preferential cleavage of Aβ_42_ by the PS1 enzyme. Transfection using PS1 resulted in an approximately 1.6 fold increase in Aβ_42_/Aβ_40_ levels [16] in cell cultures. Similarly, the APP + PS1 transgenic animals had approximately a twofold Aβ_42_/Aβ_40_ increase compared to the APP rats. The level of PS1 mRNA expression varied by tissue with the highest PS1 expression in the brain and kidney, while the heart and liver had lower expression. Tissue specific expression was also observed in the APP transgenic rats [1].

Histologic examination of the brains showed that both the APP and the APP + PS1 animals exhibited pathological abnormalities typically associated with AD. Interestingly, the APP rats had large hippocampal sections containing neurons with neurofibrillary tangles. The hippocampus of the APP + PS1 rats also contained neurofibrillary tangles, but these were more sporadic compared to what was observed in the APP rats. Interestingly, neuronal loss was much greater in the CA3 region of the APP + PS1 rats. Another difference between the hippocampus in APP and the APP + PS1 animals was that neurons in the CA1 and the CA2 regions of the APP + PS1 animals had chromatolysis. Chromatolysis is caused by axonal injury in motor neurons [18] and it has been associated with neurotoxicity and AD [5, 15, 22], but it is not reported as widely as the presence of neurofibrillary tangles or Aβ plaques.

A previously reported rat model for AD, the TgF344-AD model [6], was found to produce plaques at 15 months. The difference in plaque production between the TgF344-AD and the APP + PS1 rats could be due to the fact that the PS1 transgene in the TgF344-AD had Δ exon 9 mutation, as opposed to the L166P mutation form of the PS1 transgene that was used in these APP + PS1 rats. On the other hand, the behavioral characterization of the APP + PS1 rats in this current study revealed learning and memory deficits similar to what has been found in TgF344-AD rats. In addition, previous research has found that an AD mouse model that did not develop plaques even as late as 30 months old also showed memory deficits [9]. Consequently, the presence of Aβ plaques may not always correlate with AD severity. For example, Shankar et al. [21] showed that Aβ dimers are synaptotoxic and disrupted memory, whereas insoluble Aβ plaque cores from Alzheimer’s diseased cortex did not impair long-term potentiation. Furthermore, the number of Aβ plaques in the brain has not been found to correlate with the severity of cognitive impairments in humans or in APP and/or PS transgenic mice [3].

Wild type rats outperformed both the APP and the APP + PS1 rats during the initial learning phase of the Barnes maze test. Additionally, retention of the learned task was poorer in the APP + PS1 rats relative to both the APP and the WT rats. As stated earlier, one of the major differences between the APP + PS1 and the APP rats was that the APP + PS1 rats had neuronal loss in the CA3 region of hippocampus (Fig. 5b shows an APP + PS1 rat with very few neurons in the CA3 region). The CA1 region recodes information from the CA3 region and sets up associatively learned backprojections to the neocortex to allow subsequent retrieval of information to the neocortex [13]. The hippocampal CA3 region and the dentate gyrus also play an important role in the encoding of new spatial information and processing the geometry of the environment [12], which are both crucial to the successful completion of the task. Thus, the differences between the APP + PS1, APP, and WT rats in learning and retention in the task may be related to the differences in the pathology of the hippocampus.

## Conclusions

The addition of the PS1 transgene to the APP transgenic rat genome affected serum Aβ_42_ and Aβ_42_/Aβ_40_ levels, brain histopathology, as well as, the cognitive behavior of the double transgenic rats. Although the APP rat brain had many histopathogical abnormalities that are often seen in AD brains, their memory did not show significant impairment during the behavioral testing. In contrast, the APP + PS1 rats showed significantly reduced memory retention compared to the APP and the WT rats, in addition to, neuronal loss and necrosis in the hippocampus and brain cortex.

## Additional files

10.1186/s12868-016-0281-8 contains dataset for Northern blot analysis (see Fig. 2). PS1 mRNA level, serum Aβ42 and tissue are reported in the table.
10.1186/s12868-016-0281-8 contains dataset for serum Aβ_42_, Aβ_40_ and Aβ_42_/Aβ_40_ ratio of F1 and F2 generation transgenic rats (see Fig. 3).
10.1186/s12868-016-0281-8 contains dataset for Barnes maze (see Fig. 4). The table includes acquisition day 1, 2 and 3 latency and errors and their averages, retention latency and errors and reversal days 1, 2 and 3 latency and errors and their averages.

## Abbreviations

AD: alzheimer’s disease
Aβ: amyloid-beta
APP: amyloid precursor protein
CA: cornu Ammonis
eGFP: enhanced green fluorescence protein
IP: intraperitoneal
pLV: lentivial vector
PMSG: pregnant mares’ serum gonadotropins
PS1: presenilin 1
SSC: saline sodium citrate
SDS: sodium dodecyl sulfate
Ubi-C: ubiquitin-C
WT: wild type
WRE: woodchuck hepatitis virus post-transcriptional regulatory element
